# Tongue Pressure Modulation for Initial Gel Consistency in a Different Oral Strategy

**DOI:** 10.1371/journal.pone.0091920

**Published:** 2014-03-18

**Authors:** Sumiko Yokoyama, Kazuhiro Hori, Ken-ichi Tamine, Shigehiro Fujiwara, Makoto Inoue, Yoshinobu Maeda, Takahiro Funami, Sayaka Ishihara, Takahiro Ono

**Affiliations:** 1 Department of Prosthodontics, Gerodontology and Oral Rehabilitation, Osaka University Graduate School of Dentistry, Suita, Osaka, Japan; 2 Division of Dysphagia Rehabilitation, Niigata University Graduate School of Medical and Dental Sciences, Niigata, Niigata, Japan; 3 Texture Design Division, San-Ei Gen F.F.I., Inc, Toyonaka, Osaka, Japan; University of Toronto, Canada

## Abstract

**Background:**

In the recent hyper-aged societies of developed countries, the market for soft diets for patients with dysphagia has been growing and numerous jelly-type foods have become available. However, interrelationships between the biomechanics of oral strategies and jelly texture remain unclear. The present study investigated the influence of the initial consistency of jelly on tongue motor kinetics in different oral strategies by measuring tongue pressure against the hard palate.

**Methods:**

Jellies created as a mixture of deacylated gellan gum and psyllium seed gum with different initial consistencies (hard, medium or soft) were prepared as test foods. Tongue pressure production while ingesting 5 ml of jelly using different oral strategies (Squeezing or Mastication) was recorded in eight healthy volunteers using an ultra-thin sensor sheet system. Maximal magnitude, duration and total integrated values (tongue work) of tongue pressure for size reduction and swallowing in each strategy were compared among initial consistencies of jelly, and between Squeezing and Mastication.

**Results:**

In Squeezing, the tongue performed more work for size reduction with increasing initial consistency of jelly by modulating both the magnitude and duration of tongue pressure over a wide area of hard palate, but tongue work for swallowing increased at the posterior-median and circumferential parts by modulating only the magnitude of tongue pressure. Conversely, in Mastication, the tongue performed more work for size reduction with increasing initial consistency of jelly by modulating both magnitude and duration of tongue pressure mainly at the posterior part of the hard palate, but tongue work as well as other tongue pressure parameters for swallowing showed no differences by type of jelly.

**Conclusions:**

These results reveal fine modulations in tongue-palate contact according to the initial consistency of jelly and oral strategies.

## Introduction

In the recent hyper-aged societies of developed countries, the number of individuals with dysphagia caused by stroke, Parkinson's disease and other age-related diseases has been increasing [1], [2]. Dysphagia deteriorates activities of daily living and quality of life among the elderly [3]–[6] and causes dehydration, malnutrition and life-threatening pneumonia and suffocation [7]. The first critical point in nutritional control for dysphagic patients is to prevent aspiration. Although thickener is generally applied to liquid boluses to prevent early inflow into the pharynx and larynx [7], increased bolus viscosity may decrease the bolus transit speed, potentially increasing the risk of pharyngeal residue [8]. As for solid boluses, swallowing without sufficient mastication can again lead to residue and an increased risk of aspiration [9].

Recently, a wide variety of jelly- and mousse-type foods for patients with eating difficulties have been developed and put on the market [8], [10]. The texture of these foods is provided with consideration of masticatory/swallowing ability and oral sensation in dysphagic patients. For example, standards for hardness, cohesiveness and adhesiveness have been established in “Foods for the Elderly with Difficulty in Masticating and Swallowing” by the Ministry of Health, Labor and Welfare in Japan [11]. However the influence of food texture designed by mechanical test and sensory evaluation [12] on eating behavior (that is, oral strategy) has yet to be clarified. In dysphagic patients, not only the oral stage, but also the pharyngeal stage is often adversely affected by the collapse of coordination between tongue and jaw movements or tongue disability [13]. As many soft foods are assumed to be eaten under integrated oral strategies such as mastication and squeezing, with the comminuted or crushed bolus propelled into the pharynx by the driving force of the tongue in the final swallow [14]–[16], clarification of the influence of food texture on tongue kinetics in the oral strategy would presumably be useful in designing food texture for dysphagic patients.

Although imaging of tongue kinetics during mastication and swallowing has been performed using videofluorography [14], [15], [17]–[19], videoendoscopy [17], [18], [20] and ultrasonography [21], [22], evaluating biomechanical modulation of tongue kinetics by food texture is difficult using those modalities. Detecting the influence of change in bolus texture and amount of swallow-related tongue muscle activity using electromyography is also not easy [23]. On the other hand, the pressure produced by tongue-palate contact (tongue pressure) has received attention as a parameter representing tongue activity in the formation, transit and swallowing of the bolus, both qualitatively and quantitatively. Various investigations have examined the influence of food viscosity and consistency on tongue kinetics [24]–[31], confirming that tongue activity in swallowing is modulated by food texture. However, few studies have reported on the state of tongue pressure generation during the food-processing period before swallowing. This might be because of the technical difficulty of measuring tongue pressure using intra-oral devices that might interfere with the conduction of oral strategy such as mastication and squeezing.

We have developed a novel tongue pressure measurement system [32] that can be applied to young and elderly healthy subjects and stroke patients to investigate tongue kinetics during swallowing [33]–[37]. The ultra-thin sensor sheet in this system can be attached directly to the hard palate and records the state of tongue-palate contact under nearly natural conditions without interfering with occlusal contact, and is considered suitable for investigating the influence of food texture on tongue kinetics. The current study therefore investigated the influence of the initial consistency of a jelly that is widely used in dysphagia rehabilitation on tongue pressure production with different oral strategies (squeezing/mastication). The tongue pressure measurement system was used to test the hypothesis that tongue pressure production before and during swallowing would differ according to both the initial consistency of the jelly and the type of oral strategy for size reduction.

## Materials and Methods

### Subjects

Subjects in the present study were eight healthy volunteers (four men, four women; mean age, 27.2±1.7 years) without tooth loss except for the third molar, and with no history of dysphagia, temporomandibular disorder or orthodontic treatment. All subjects provided written informed consent prior to enrolment. The protocol of this study was approved by the ethics committee of Osaka University Graduate School of Dentistry (H21-E32).

### Preparation of jelly samples

Jellies created using a mixture of deacylated gellan gum and psyllium seed gum (SAN SUPPORT G-1014; San-Ei Gen F.F.I., Toyonaka, Japan) were prepared with three different consistencies: 1 w/w% for soft jelly; 1.8 w/w% for medium jelly; and 2.8 w/w% for hard jelly (Table 1). In terms of jelly structure, gels work well not only as an experimental material due to the easiness of rheological characterization and high reproducibility of quality, but also as a food matrix themselves for dysphagia products. Soft and hard jellies corresponded to criterion II of “Foods for the Elderly with Difficulty in Masticating and Swallowing” issued by Japanese Ministry of Health and Welfare [11], whereas medium jelly corresponded to criterion I of the same regulation, as determined by two-bite compression of jelly samples (diameter, 40 mm; height, 15 mm) at a table speed of 10 mm/s using a 20-mm-diameter flat aluminum-plunger, as often found in texture profile analysis [38]. Soft jelly was also categorized into group 4 (unnecessary to chew) of the “Universal Design Foods” issued by the Japan Care Food Conference [39], whereas medium and hard jellies were categorized into group 3 (squeezable with the tongue) of the same regulation, as determined by the same method described above. None of the types of jelly dissolved or melted in the mouth on mixture with saliva at body temperature [40]. The variations of jelly consistency used were based on the experimental design for investigating tongue pressure modulation in both types of food oral strategy: squeezing, and mastication.

**Table 1:** Instrumental texture properties of gel samples.

Consistency	Concentration (w/w%)	Hardness (N/m^2^)	Adhesiveness (J/m^3^)	Cohesiveness
**Soft**	1.0	1618±68	44±2	0.32±0.01
**Medium**	1.8	5738±85	112±9	0.32±0.02
**Hard**	2.8	12317±20	192±14	0.38±0.01

The three mechanical parameters (hardness, adhesiveness, and cohesiveness) were determined by two-bite compression of jelly samples (diameter, 40 mm; height, 15 mm) at a table speed of 10 mm/s using a 20-mm-diameter flat aluminum plunger, and values are given as mean ±SD.

### Tongue pressure measurement

**Figure 1:**
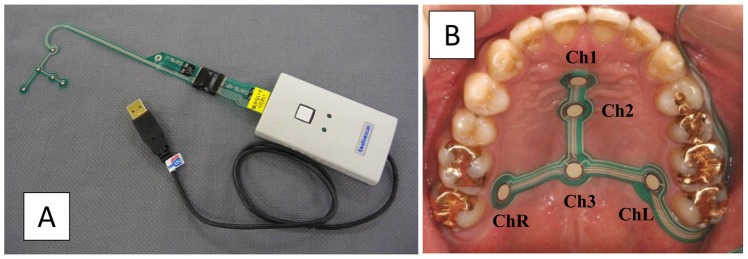
The system for measuring tongue pressure. A) Swallow scan; Nitta, Osaka, Japan, B) Sensor sheet with five measuring points (Chs.1–5) attached to the hard palate.

The tactile sensor system Swallow-Scan (Nitta, Osaka, Japan), with a 0.1-mm-thick sensor sheet for measuring tongue pressure, was used in this study (Fig. 1) [32]. A T-shaped sensor sheet with five measuring points was designed based on our previous studies [41], [42] using electric pressure sensors; three measuring points (Chs.1–3) were placed along the median line (Ch.1 set at the anterior-median part, Ch.2 at the mid-median part, Ch.3 at the posterior-median part), and two sensors [Chs.L (left position) and R (right position)] were situated in the posterior-circumferential parts of the hard palate. Sampling frequency was 100 Hz. Positions of the sensor sheet were defined on the basis of anatomical landmarks such as the incisive papillae and the hamular notch. Ch.1 was positioned 5 mm posterior to the incisive papillae. Ch.2 was placed at the anterior one-third, and Ch.3 at the posterior one-third on the vertical line passing through the center of bilateral hamular notches. Ch.L and Ch.R were set at the posterior one-third on the line connecting the incisive papillae and the hamular notch, respectively. A small, medium, or large sensor sheet was selected for each subject according to the size of the hard palate.

The sensor sheet was attached to the palatal mucosa directly with a sheet-shaped denture adhesive (Touch Correct II; Shionogi, Osaka, Japan) during the recording of tongue pressure. After placement of the sensor sheet on the palate, calibration was performed by applying negative pressure on the cable of the sensor sheet using a vacuum pump. Before measuring tongue pressure, the sensor sheet was confirmed to be properly attached to the subject's palate with no interference with occlusion and no discomfort. For recording the timing of laryngeal movement during swallowing, a microphone (JM0116; Ono Sokki, Kanagawa, Japan) was attached externally at the level of the inferior border of the cricoid cartilages and recorded swallowing sounds.

Tongue pressure was recorded with the subject sitting on a chair in an upright position. The head was supported by the head-rest of the chair so the Frankfort plane was parallel to the ground, with the feet touching the ground. A 5-ml portion of jelly was inserted into the subject's mouth by the investigator. After the jelly was introduced into the subject's mouth, the subject applied one of the two oral strategies. The first oral strategy, “Squeezing”, involved swallowing after squeezing the jelly between the tongue and palate, without mastication. The second, “Mastication”, involved swallowing the jelly after free mastication. Squeezing and Mastication were performed by each subject three times for the three consistencies of prepared jelly, provided in random order. Tongue pressure and swallowing sounds were transmitted in real time to a personal computer, which stored the data.

### Data analysis

**Figure 2:**
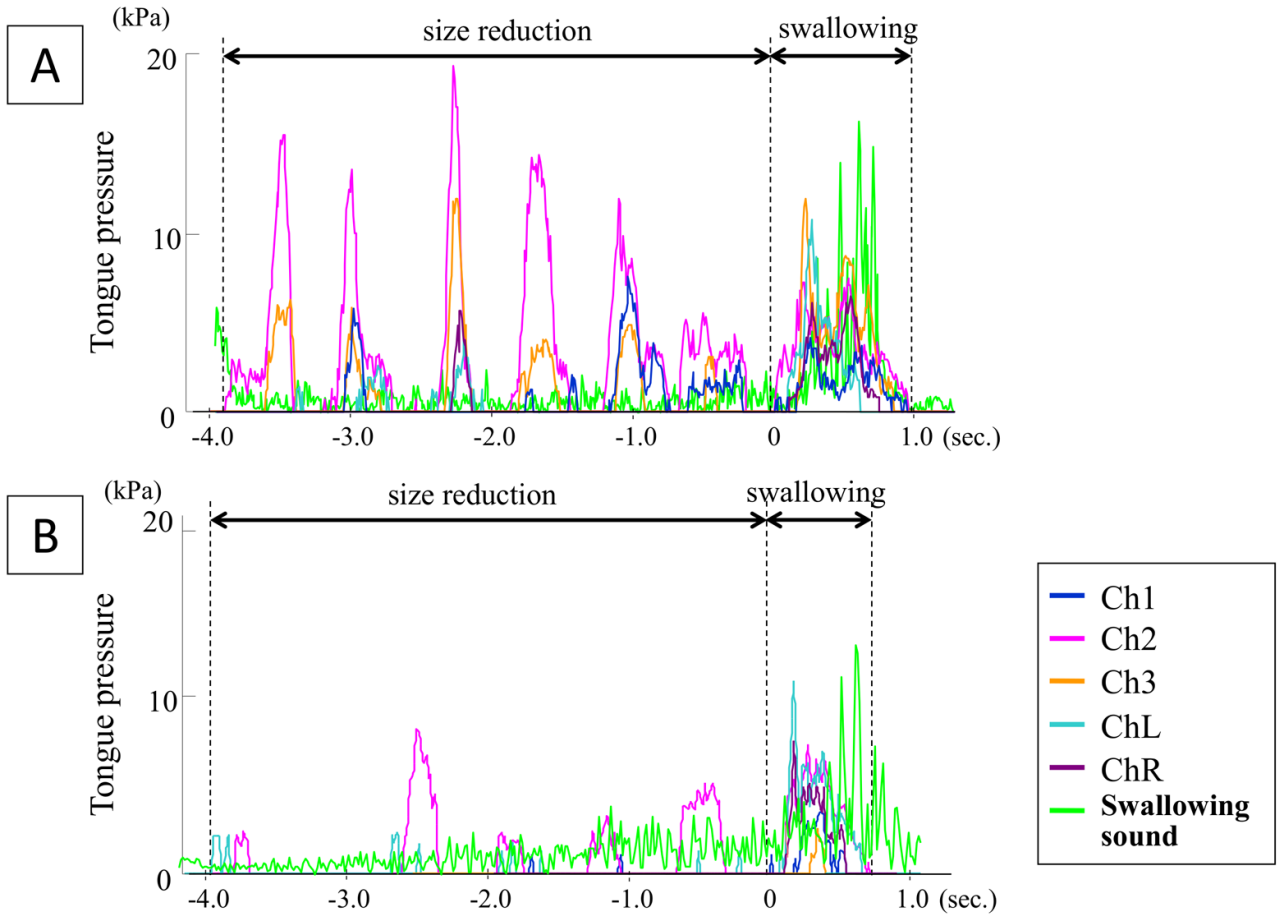
Representative waves of tongue pressure during oral processing and final swallow. A) Squeezing; B) Mastication.

Typical waveforms during Squeezing and Mastication are illustrated in Figure 2. For differentiating swallowing pressure wave from squeezing/mastication pressure waves, we referred to the peak of swallowing sounds, which synchronized well with swallowing pressure. Because the tongue comes into contact with Ch.1 earlier than with other measuring points, the time of onset at Ch.1 was recognized as the beginning of swallowing pressure. Maximal magnitude of tongue pressure (representing the maximal strength of tongue-palate contact), duration of tongue pressure (representing the sequential length of tongue-palate contact) and integrated value of tongue pressure (representing the total amount of tongue work for size reduction and swallowing) in both strategies were calculated for each channel. Variations were compared between jellies of different consistencies and between different oral strategies. Repeated one-way analysis of variance was carried out for statistical analysis, and when a significant difference was identified, Bonferroni's multiple comparison was performed. Values of P<0.05 were considered statistically significant.

## Results

### Maximal magnitude of tongue pressure (Fig. 3)

**Figure 3:**
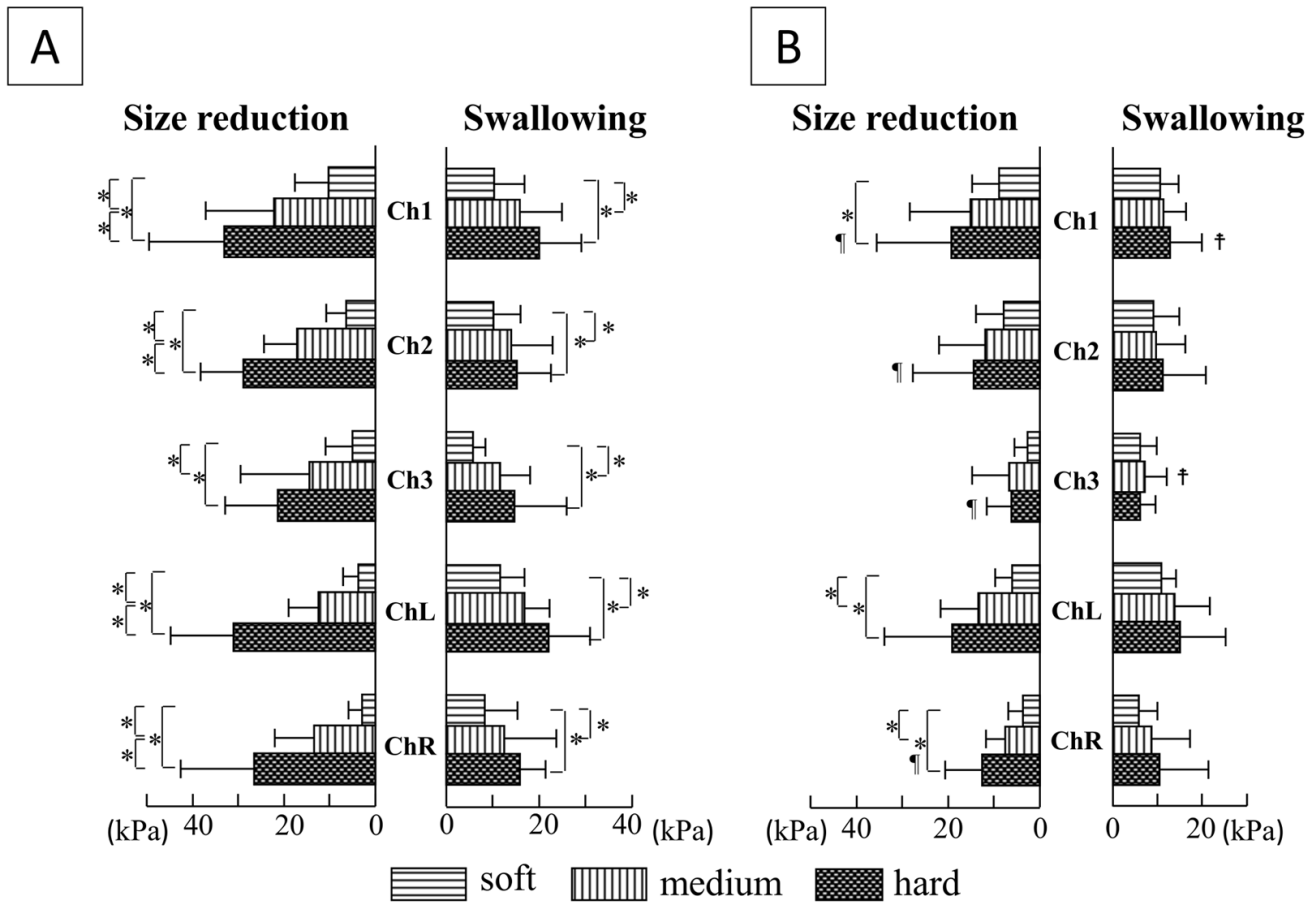
Comparisons of maximal magnitude of tongue pressure among the three hardnesses of jelly, and between oral strategies. A) Squeezing; B) Mastication. *P<0.05. ¶: Maximal magnitude of tongue pressure for size reduction was smaller in Mastication than in Squeezing (P<0.05), ‡: Maximal magnitude of tongue pressure for swallowing was smaller in Mastication than in Squeezing (P<0.05).

Maximal magnitude of tongue pressure for size reduction in Squeezing increased significantly in a stepwise manner with increasing initial consistency of the jelly at each measuring point except Ch.3, which showed the same tendency as other points. Maximal magnitude of tongue pressure for swallowing in Squeezing also tended to increase at each measuring point as the initial consistency of jelly increased.

Maximal magnitude of tongue pressure for size reduction in Mastication increased with the increase in initial consistency of the jelly at Ch.1 and Chs.L and R. However, maximal magnitude of tongue pressure for swallowing in Mastication was unchanged at any measuring point, even if the initial consistency of the jelly increased.

When comparing the same measuring point with the same initial consistency of jelly between oral strategies, maximal magnitude of tongue pressure for size reduction was larger in Squeezing than in Mastication with hard jelly at Chs.1–3 and R, and maximal magnitude of tongue pressure for swallowing was smaller in Mastication than in Squeezing at Ch.1 with hard jelly and Ch.3 with medium jelly.

### Duration of tongue pressure (Fig. 4)

**Figure 4:**
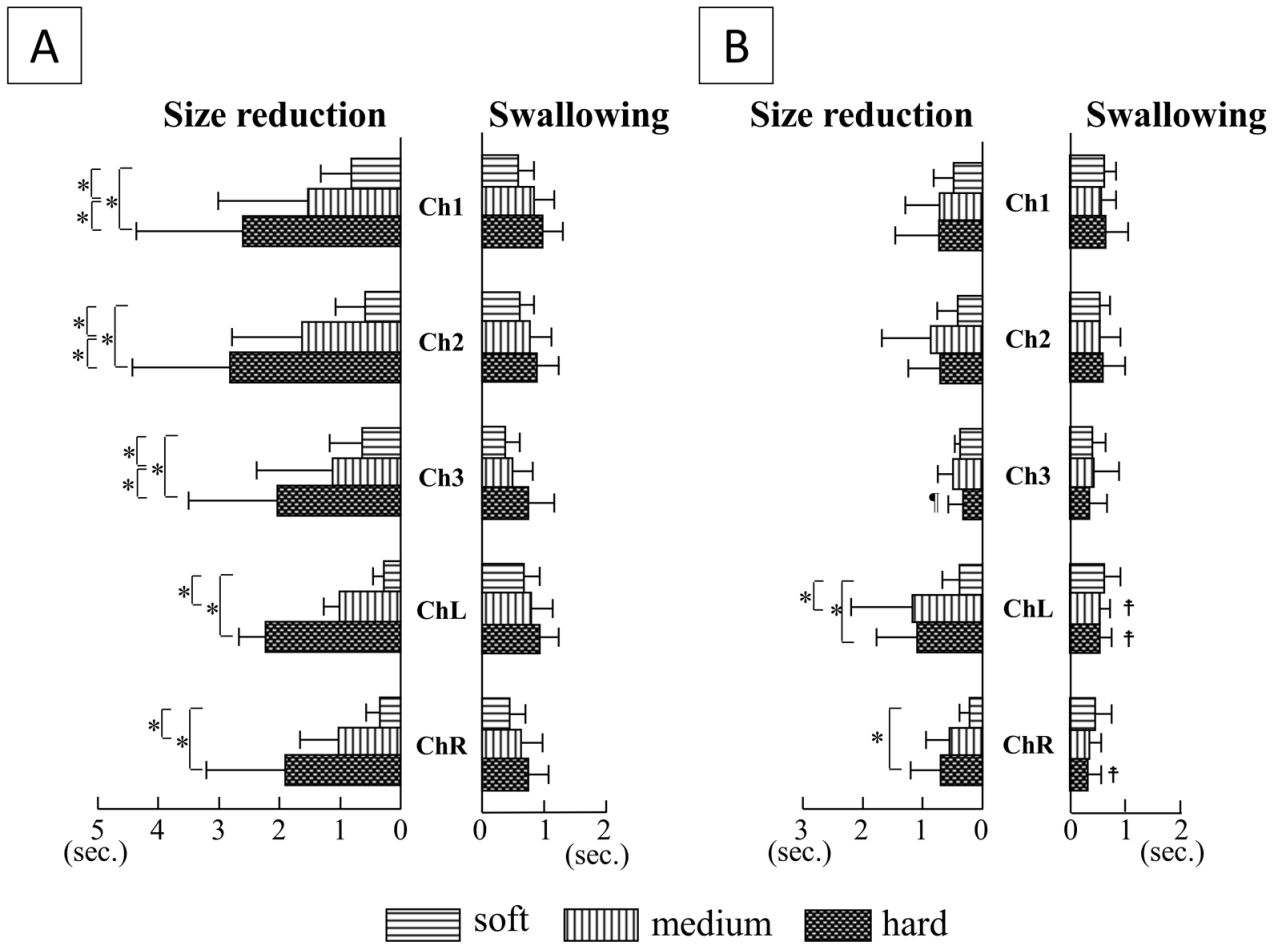
Comparisons of duration of tongue pressure among the three hardnesses of jelly, and between oral strategies. A) Squeezing; B) Mastication. *P<0.05. ¶: Duration of tongue pressure for size reduction was shorter in Mastication than in Squeezing (P<0.05). 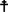: Duration of tongue pressure was longer in Mastication than in Squeezing (P<0.05).

Total duration of tongue pressure for size reduction in Squeezing increased in a clear step-wise manner as the initial consistency of jelly increased at Chs.1–3, and the same tendency was found at Chs.L and R. On the other hand, duration of tongue pressure for swallowing in Squeezing showed no differences according to initial consistency of jelly at any measuring points.

Although the total duration of tongue pressure for size reduction in Mastication at Chs.1–3 showed no influence of the initial consistency of jelly, values at Chs.L and R increased with increasing consistency of jelly. On the other hand, no differences in duration of tongue pressure for swallowing according to initial consistency were seen at any measuring points in Mastication.

When comparing the same measuring point and the same consistency between oral strategies, duration of tongue pressure for size reduction was larger in Squeezing than in Mastication with hard jelly at Ch.3, and the duration of tongue pressure for swallowing was shorter in Mastication than in Squeezing at Ch.L with medium and hard jellies and at Ch.R with hard jelly.

### Total integrated value of tongue pressure (Fig. 5)

**Figure 5:**
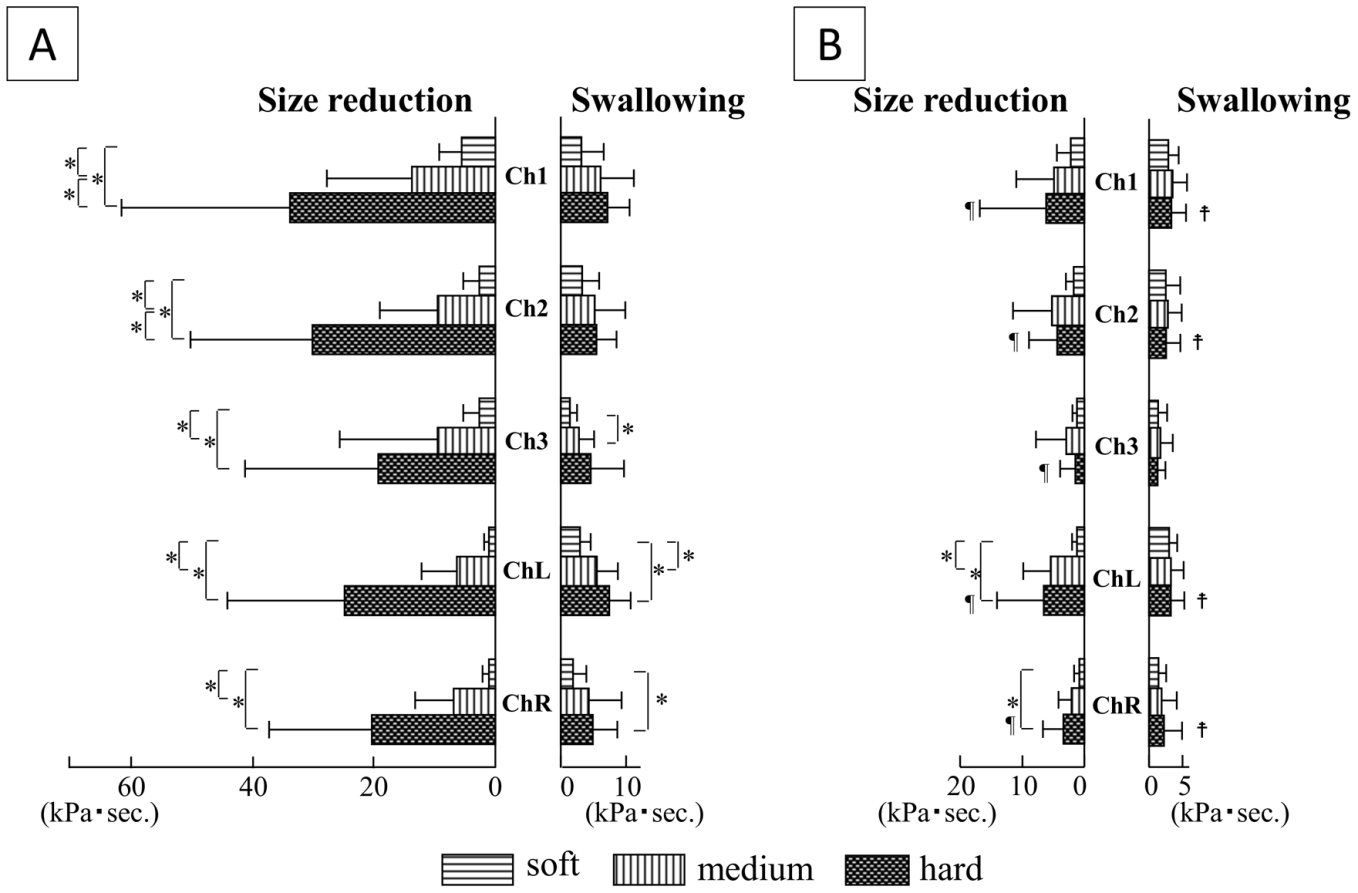
Comparisons of integrated value of tongue pressure among the three hardnesses of jelly, and between oral strategies. A) Squeezing; B) Mastication. *P<0.05. ¶: Integrated value of tongue pressure for size reduction was smaller in Mastication than in Squeezing (P<0.05), 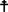: Integrated value of tongue pressure was larger in Mastication than in Squeezing (P<0.05).

Total integrated value of tongue pressure for size reduction increased in Squeezing in a clear, step-wise manner at Chs.1 and 2, and showed the same tendency at all other measuring points (Chs.3, L and R). Integrated pressure values for swallowing tended to be increased in Squeezing with increasing initial consistency of the jelly at Chs.3, L and R.

Total integrated value of tongue pressure for size reduction in Mastication tended to increase with increasing initial consistency of jelly at Chs.L and R. As for the integrated value of tongue pressure for swallowing in Mastication, no influence of jelly consistency was seen at any measuring points.

Comparing the same measuring point and the same consistency between oral strategies, total integrated value of tongue pressure for size reduction was larger with hard jelly at all measuring points, and that for swallowing was smaller in Mastication than in Squeezing at all measuring points except Ch.3 with hard jelly.

## Discussion

The present results revealed fine modulations in tongue-palate contact with changes in the initial consistency of jelly and oral strategy. Although numerous studies have reported that tongue activity is modulated by the viscosity of liquids and the consistency of solid/semi-solid foods [25]–[27], [30], [31], [43], [44], details of the influence of oral strategy do not appear to have been described previously. The sensor sheet system used in this study offers methodological advantages for measuring tongue pressure at multiple points under near-natural conditions. Previous studies applied pressure sensors installed in a palatal plate, so coverage of the palate by this plate intercepted the sensory stimuli and required an adaptation period [45], [46]. Our sensor sheet system enabled palatal sensation to be retained, because the area of coverage was quite limited. As the attached cable was designed to avoid interrupting the occlusal contact during chewing and swallowing, modulation of tongue pressure based on the sensory input from gum, palatal mucosa and periodontal ligaments [47], [48] might occur in each subject. In addition, we analyzed the state of tongue pressure generation using three parameters: maximal magnitude; duration; and total integrated value. These values could describe how tongue work (total integrated value) for the generation of pressure for oral processing was modulated by changes in magnitude and duration of pressure.

In Squeezing, the tongue performed more work for size reduction with increased initial consistency of jelly by modulating both magnitude and duration of tongue pressure over a wide area of hard palate, but tongue work for swallowing increased at the posterior-median and circumferential parts by modulating only the magnitude of tongue pressure. The first part of these results shows that both fine and wide-range tuning of the magnitude and duration of tongue-palate contact can be performed for size reduction in Squeezing, which is a voluntary movement. The later parts suggest that bolus texture just before swallowing may differ among each jelly irrespective of tongue pressure modulation during size reduction, and such differences require the modulation of swallowing pressure. The finding that duration of tongue-palate contact is not involved in this modulation appears logical, because the sequential pattern should be firm in reflex movements such as swallowing.

On the other hand, in Mastication, the tongue performed more work for size reduction according to increased initial consistency of the jelly by modulating both magnitude and duration of tongue pressure, mainly in the posterior part of the hard palate. However, tongue work and other tongue pressure parameters for swallowing showed no differences among types of jelly. These results suggest that Mastication is a very effective oral strategy for forming a bolus of a certain texture before swallowing, irrespective of variations in initial consistency of the jelly. Furthermore, the greater tongue work for size reduction and swallowing seen in Squeezing with hard jelly than in Mastication suggests that the burden on the tongue is increased in Squeezing with hard jelly.

As a possible background to these results, rheological and tribological properties of the food bolus may need to be changed according to the oral strategy for size reduction of Squeezing or Mastication, due to differences in the particle size of the bolus and miscibility with saliva [49]. If these food properties before consumption are the same, the tongue pressure required for swallowing can be differentiated according to the oral strategy for size reduction. Based on technical information from the manufacturer, gelling agents used in this study for the preparation of jellies were optimized in texture and designed for dysphagia application by balancing elasticity (mainly from deacylated gellan gum) and viscosity (mainly from psyllium seed gum). At relatively high concentrations of gelling agent, viscosity can predominate over elasticity, providing deformability in jellies [50]. Jelly samples of relatively high concentrations cannot be sufficiently reduced in size by the modulation of tongue pressure alone in Squeezing. We anticipate that Mastication would allow the formation of a bolus that is swallowable at lower tongue activity compared to Squeezing, due to the higher size-reduction efficiency of Mastication.

These speculations are supported by the current results that tongue pressure generation differed at certain measuring points between Squeezing and Mastication. Meanwhile, maximal magnitudes of tongue pressure for swallowing in Squeezing at the anterior-median part (Ch.1) with hard jelly and at the posterior-median part (Ch.3) with medium jelly were larger, and duration of tongue pressure for swallowing was longer in Squeezing than in Mastication at posterior-circumferential parts (Chs. L and R). The increase in maximal magnitude in Squeezing at Chs.1 and 3 was attributed to hard jelly tending to be crushed by pushing the tip of the tongue against the anterior hard palate (around Ch.1) [43] and the bolus was enveloped between the posterior hard palate (around Ch.3) and dorsum, then transferred into pharynx [14]–[16]. In addition, a reason why the duration of tongue pressure was prolonged at Chs.L and R might be that heterogeneous particle sizes resulted in differences in the destination period of the bolus enveloped between the tongue and palate in cases of relatively high-density gel agent that could not be crushed by Squeezing. Gels are differentiated from hard and non-deformable foods like peanuts and raw carrots, in that gels can be swallowed even if particles of relatively large size exist in the bolus. Peanuts and raw carrots can be swallowed, on the other hand, when cohesiveness (internal binding force) of the bolus increases upon mastication as a result of the formation of small and homogeneous (in size and shape) particles [51]. These differences are attributed to the deformability of gels and are characteristic to gels as viscoelastic bodies.

The high capacity of modulation in tongue pressure generation that was found in the healthy young subjects of the current study was not necessarily expected for elderly individuals. Hard jellies required greater tongue work for pressure generation in size reduction and swallowing in Squeezing than in Mastication. Therefore, for the elderly with poor chewing ability because of tooth loss and/or muscle weakness, the texture of soft food should be carefully decided by considering the increased burden on the tongue kinetics when the individual ingests food by Squeezing as a compensatory strategy for Mastication.

## Conclusion

A series of tongue pressure measurements during two oral strategies –Mastication and Squeezing–revealed that the tongue modulated tongue work for generating contact pressure against the hard palate where the duration and maximal magnitude of tongue pressure increased with increasing initial consistency of jelly. Swallowing pressure was modulated by the initial consistency of jelly in Squeezing, but was kept constant irrespective of consistency in Mastication. Our results clearly show that the initial consistency of jelly and oral strategy influence the modulation of tongue-palate contact through the biomechanics of oral processing, in turn suggesting the possibility of designing food textures for individuals with masticatory and/or swallowing disturbances using tongue pressure measurements.
